# P-246. Abstract Title: Neuropsychiatric Outcomes Associated With PI- vs. INSTI-Based Regimens in People With HIV: A Propensity-Matched Cohort Study

**DOI:** 10.1093/ofid/ofaf695.468

**Published:** 2026-01-11

**Authors:** Aimalohi Okpeku, Elvin T Price

**Affiliations:** Virginia Commonwealth University, Richmond, VA; Victor A. Yanchick Associate Professor, Director of the Geriatric Pharmacotherapy Program. Department of Pharmacotherapy &Outcomes Science. Virginia Commonwealth University., Richmond, Virginia

## Abstract

**Background:**

Neuropsychiatric symptoms (NPS), including anxiety, depression, and cognitive impairment, are increasingly recognized as adverse outcomes among people living with HIV (PLWH). As antiretroviral therapy (ART) use expands and treatment duration increases, concerns have emerged about neuropsychiatric effects of specific ART regimens. This study compared the risk of NPS among users of protease inhibitor (PI)-based and integrase strand transfer inhibitor (INSTI)-based ART.

**Methods:**

We conducted a retrospective cohort study using the TriNetX Research Platform, a global, federated, de-identified real-world data system. Adults (≥18 years) with HIV prescribed PI- or INSTI-based regimens between January 1, 2015, and December 31, 2022, were included. Patients with prior NPS were excluded. Before matching, 5,063 PI and 11,788 INSTI users were identified. Propensity score matching (1:1) on demographics and comorbidities produced balanced cohorts (n = 5,058 per group). Kaplan–Meier analysis evaluated risk of anxiety, depression, and cognitive impairment at 12, 18, 24, and 36 months.

**Results:**

At each time point, the PI group had a significantly higher probability of remaining free from anxiety:

12 mo: 94.7% vs. 91.5% (p = 0.0002; RR = 0.74, 95% CI [0.63–0.87])

18 mo: 91.9% vs. 89.9% (p = 0.00014; RR = 0.80 [0.69–0.92])

24 mo: 90.3% vs. 87.5% (p = 0.0004; RR = 0.81 [0.71–0.92])

36 mo: 88.9% vs. 86.5% (p = 0.0002; RR = 0.84 [0.75–0.93])

No significant differences were observed between the two groups for depression or cognitive impairment at any follow-up point. Subgroup analysis of individuals aged >65 years showed no significant differences in neuropsychiatric risk between treatment groups.

**Conclusion:**

This large- scale real- world study among adults with HIV showed that PI based regimens were associated with a significantly reduced risk of anxiety at 12,18, 24, 36 months compared with INSTI based regimens. These findings highlight the importance of considering neuropsychiatric events when selecting ART regimens. Further research is needed to understand these associations and potential confounding factors.

**Disclosures:**

All Authors: No reported disclosures

